# Epidemiology and high incidence of metallo-β-lactamase and AmpC-β-lactamases in nosocomial *Pseudomonas aeruginosa*

**DOI:** 10.22038/IJBMS.2021.57293.12748

**Published:** 2021-10

**Authors:** Maria Muddassir, Sadaf Munir, Almas Raza, Ahmad Basirat, Muddassir Ahmed, Umar Farooq, Syed Shoaib Ahmed, Syed Zeeshan Haider Naqvi

**Affiliations:** 1 Institute of Molecular Biology & Biotechnology (IMBB), The University of Lahore, Defence road campus, Lahore, Pakistan; 2 Department of Pathology, Combined Military Hospital, Lahore Cantt, Pakistan; 3 Department of Respiratory Medicine, Tallaght University Hospital, Dublin, Ireland; 4 M. Islam Medical College, Gujranwala, Pakistan; 5 Al-Aleem Centre for Advanced Studies and Research, Gulab Devi Educational Complex, Lahore, Pakistan

**Keywords:** Antibiotic resistance, Beta-Lactamases, Infections, MDR genes, Pseudomonas aeruginosa

## Abstract

**Objective(s):**

Isolates producing metallo-β-lactamase (MBL) have a significant impact on therapeutic and diagnostic layouts, plus their increased frequency has been reported globally. Determination of incidence of clinical isolates of *Pseudomonas aeruginosa* that are capable of producing MBL and AmpC-β-lactamases making them resistant to imipenem and cefoxitin.

**Materials and Methods:**

Out of 1159 collected samples of urine, wound swabs, blood, tissue, and pus, the isolation rate of *P. aeruginosa* in the period of March 2020 to February 2021 was 22.0% (255/1159). Bacterial strains that were resistant towards imipenem were further processed for detecting the β-lactamase group of genes followed by statistical analysis of risk factors done based on clinical sample, gender, plus department of sample collection.

**Results:**

The percentage of resistance against imipenem was found to be 53%. Out of 135 strains, phenotypic tests revealed MBLs incidence to be 61.5% by combination disc test and 81.5% by Modified Hodge test (MHT). Frequencies of blaIMP-1, blaVIM, blaSHV, blaTEM, and blaOXA genes were calculated to be 13%, 15%, 32%, 43%, and 21%, respectively. Co-expressions of blaMBLs (blaVIM and blaIMP-1) plus blaESBL (blaSHV, blaOXA, blaTEM) were detected using simplex and multiplex PCR. blaTEM, blaSHV, and blaOXA co-existed in 7.5% of clinical isolates. 5.5% of the isolates exhibited simultaneous expression of MBL/ESBL genes. 15% of the isolates resistant to cefoxitin were positive for the blaAmpC gene (17/114).

**Conclusion:**

This is a pioneer report from Pakistan that concomitantly presents expression of blaVIM and blaIMP-1 with blaTEM, blaOXA, blaSHV, and blaAmpC in isolates of *P. aeruginosa*.

## Introduction

The prevalence and high frequency of serious infections caused by pathogens that produce β-lactamases is a significant threat to antibiotic treatment. The extensive usage of carbapenems has led to the development of resistance against this class of antimicrobial agents (1–3). Resistance against carbapenems is a threat due to the fact that the treatment options for resistant microbes are extremely limited (4, 5). The multiple resistance mechanisms against imipenem include the AmpC enzyme plus the alterations to membrane porin along with efflux pumps up-regulation (5, 6). Overproduction of enzymes of ESBL-type has been reported previously (5, 7). The second phenomenon that plays a part in the development of antimicrobial resistance is hydrolysis of carbapenems by enzyme carbapenemases (7–9).

The origin of emerging of carbapenemases plus extended-spectrum-β-lactamases (ESBLs) was different. Nevertheless, genes associated with both carbapenemases and ESBL are evident as reported by various studies. These studies have reported co-existence of ESBL genes in clinical isolates that have been found to produce MBL (10). Despite the quite uncommon origination of the two essential groups of β-lactamases, blaMBL and blaESBL nevertheless seem to be quite associated with each other (10). From the list of carbapenemases, the Metallo-β-lactamase (MBLs) hold immense significance for this part of the world since the emerging of newer variants of MBLs like New Delhi MBL (NDM) (11) plus different variants of IMP from the subcontinent. In accordance with the Ambler classification system, MBLs, MBLs belong to class B carbapenemases (12). blaNDM, blaVIM, and blaIMP hold utmost importance. Mobile plasmids carry MBL gene clusters and they are present in a number of clinically significant microbes (13, 14). Additionally, oxacillinases belong to the class D carbapenemases. These constitute serine β-lactamases that have widely been found in association with the epidemics related to carbapenem-resistant Enterobacteriaceae. Globally, almost 37 different types of IMP carbapenemases have been reported to date (15), amongst which IMP-1 has been the first carbapenemase that was reported in 1991 in Japan (16). Type IMP-4 enzymes that were initially discovered in the 2000s in Hong Kong (17), were then discovered to be the causative carbapenemase for an epidemic in Melbourne 2005 (18). The outbreak in Australia was later attributed to the spreading of resistance genes to members of *Enterobacteriaceae* from *Pseudomonas aeruginosa*. About 20 subtypes of different IMP enzymes are associated with *P. aeruginosa *infections worldwide (19). Initial reports of resistance because of enzyme VIM-1 were from Verona, Italy in 1999 (20). Reports of developing resistance due to VIM-2 have increased in Asia, Europe, Africa, and America (21). Four new variants of VIM have been recently reported by a global surveillance study (22). Discovery of NDM-1 was reported from New Delhi in 2009 (23). *Escherichia coli* and *Klebsiella pneumoniae* have been found to produce NDM-1 extensively as shown by various reports from Japan, China, Syria, and the European countries (24–27). 

Studies conducted in Pakistan, UK, and India have shown the spread of the NDM-1 carbapenemase gene via horizontal gene transfer (28). The increase of resistance against carbapenems especially in Asian countries is quite evidenced since reporting has revealed that resistance against imipenem has spiked from 40% in Vietnam (29) to about 20% in the Philippines (30). Unfortunately, the resistance pattern of MBLs has not quite been extensively studied in Pakistan. Reports of resistance against imipenem were rarely available in Pakistan before 2000 (31). Resistance to carbapenems in *P. aeruginosa *was later reported only in Karachi and Lahore (32, 33). Another study conducted in Rawalpindi stated that almost 78% of clinical isolates were detected to be producers of MBLs and majorly the production was found in *P. aeruginosa* (34). Quite a limited number of studies and reports are available from Pakistan that entail the basis of molecular analysis of genes that can possibly be acquired by the isolates that are carbapenem-resistant. The current study thus aims for determination of the incidence of MBLs through both phenotypic and genotypic analysis. Furthermore, this study was undertaken to possibly detect various variants of the gene that can be causative for resistance against carbapenems. In accordance with our knowledge, this report is the first from Pakistan that has been conducted based on the co-existence and molecular epidemiology of blaMBLs and blaESBLs plus AmpC beta-lactamase.

## Materials and Methods

***Study design***

Bacterial samples were processed at the Pathology laboratory of Jinnah hospital Lahore from March 2020 to February 2021. The Ethical Committee of University of Lahore (Ref # IMBB/UOL/20/138) has approved this research project. 

***Bacterial isolates***

In total, 1159 clinical specimens were collected from in-hospital patients. All these samples were processed by isolating and identifying pathogens in accordance with CLSI guidelines procedures (CLSI, 2019). *P. aeruginosa* was isolated from 255 of the clinical specimens which were further screened for resistance to various antibiotic drugs including imipenem and ceftazidime. Out of a total of 255 isolates, 145 isolates were from females and 110 were obtained from males (Table 1). Age group 40–49 years showed the highest isolation rate amongst all age groups (Table 2). Based on the pattern of antibiotic susceptibility, 135 isolates that were resistant to imipenem and 153 isolates that were resistant against ceftazidime were analyzed using molecular methods. Isolates of *P. aeruginosa *were identified by characteristics of bacterial culture plus Gram staining along with conventional biochemical testing. Furthermore, *P. aeruginosa *was identified using the API20NE identification strips (bioMerieux, France). The strains that were identified were then stored in 30% glycerol broth at −70 °C. Department-wise isolation of *P. aeruginosa *was surgery n=94(36.8%), medicine n=66(25.9%), orthopaedics n=34(13.3%), ICU n=29(11.4%), ENT n=14(5.5%), and gynaecology n=18(7.0%) (*P*≤0.001). Sample-wise isolation of *P. aeruginosa *was wound swabs n=89(34.9%), urine n=71(27.8%), sputum n=35(13.7%), blood n=30(11.7%), pus n=18(7.05), and tissue n=12(4.7%) (Table 4). 

***Antimicrobial susceptibility testing***

The Kirby-Bauer method was performed for testing antimicrobial susceptibility of clinical isolates of *P. aeruginosa *(35). This was performed on Mueller-Hinton agar plates (Oxoid) in accordance with CLSI 2019 (Clinical and Laboratory Standards Institute 2019) recommendations. The antibiotics that were employed for screening cultures were specific for Gram-negative bacteria. Antibiotic discs used were amikacin (30 µg), piperacillin/tazobactam (100 µg), gentamicin (10 µg), cefoperazone/sulbactam (75–10 µg), imipenem (10 µg), aztreonam (10 µg), ciprofloxacin (5 µg), meropenem (10 µg), ceftazidime (30 µg) and cefoxitin (30 µg). Susceptibility testing results were further used for calculation of multiple antibiotic resistance index (MAR) of the collected isolates of *P. aeruginosa *for estimation of the trends of drug resistance plus emergence of novel resistant bacterial isolates.

***MBLs- Phenotypic detection***

MBLs were phenotypically identified employing three tests in accordance with CLSI guidelines (CLSI, 2019). The combination disc synergy test (CDST) was done by employing a disc of imipenem alone plus IMP/EDTA disc according to the method suggested by Wadekar *et al*. (36). This was followed by the Modified Hodge test (MHT) in accordance with the methodology by Kumar *et al*. (37). The results were then interpreted according to a criterion that has been stated in CLSI 2019. Antibiotics used were from Oxoid, Inc. (Canada). The E-strips incorporated with IMP/EDTA plus IMP alone were further utilized for detection of MBLs in accordance with the manufacturer’s instructions (Liofilchem®). 

***MBLs-Molecular characterization-Preparation of DNA template for PCR***

According to the methodology described previously, the template DNA was extracted from clinical isolates (38). Briefly, a few colonies of bacterial isolates were suspended in 300 µl of distilled water and then boiled for about 10 min. This emulsion of bacterial cells was then centrifuged at 12000 rpm for 10 min and the supernatant collected was utilized as a template for the processing of PCR amplification. Mixture for PCR included 200 µM dNTP, 10 pM for primer, 1.5 mM MgCl_2, _50 ng DNA templates, and 0.5 U Taq Polymerase. The final volume was 25 µl. The products of PCR were then analyzed at 70 V for 30 min. The gel used was 1.5 w/v agarose plus Ethidium bromide 500 µg/100 ml. 

***Detection of ESBL/ MBL genes***

Singleplex PCR was used for confirmation of the positive MBLs isolates on the basis of phenotype detection. Primer sequences that were employed for detecting blaIMP-1, blaOXA, blaSHV plus blaTEM genes have been previously reported (39). Isolates of *P. aeruginosa *were then screened for blaVIM and blaIMP-1 genes by employing the singleplex PCR by primers that have been reported previously (40, 41). Mixture for PCR included 200 µM dNTP, 10 pM for primer, 1.5 mM MgCl_2, _50 ng DNA templates, and 0.5 U Taq Polymerase. The final volume was 25 µl. Obtained products of PCR were then analyzed at 70 V for 30 min. The gel used was 1.5 w/v agarose plus Ethidium bromide 500 µg/100 ml. Conditions for amplification were an initial denaturation at 95 °C, 35 cycles of 1 min denaturation at 95 °C, 1.5 min annealing, extension for a further 1 min, and then followed by a final extension for 10 min at 72 °C. The concentration of Mg was maintained between 1 and 1.5 mM. Presence of the blaAmpC gene in isolates was by PCR amplification of 1063 bps for the Amp-C beta-lactamase gene (Table 3).

***Statistical analysis***

Statistical analysis of the demographic data was performed using SPSS version 20. Proportions of multidrug resistance genes were analyzed using the chi-square test. A *P*-value of < 0.05 was considered to be statistically significant. The associations among department of the sample, type of sample, and gender were further calculated. 

## Results

***The distribution of clinical isolates***

The prevalence of *P. aeruginosa* was 22.0% (255/1159). The current study reports the patterns of resistance of clinical isolates that were resistant to imipenem and ceftazidime. The incidence of MBL production in the isolates of *P. aeruginosa* was determined. Furthermore, significant gene variants that can possibly be associated with the MBL phenotype were also analyzed. Department of clinical isolate plus type of isolate were the salient features of statistical analysis. Total clinical specimens were 1159 out of which isolates of *P. aeruginosa *were 255. Among the collected samples, 53% exhibited resistance to imipenem and 60% exhibited resistance against ceftazidime. A high frequency of *P. aeruginosa* was isolated from in-hospital patients in the age group of 40-49 years with females in total having a higher number of infections with *P. aeruginosa* (*P*≤0.001). 

***MBL isolates-antibiotic susceptibility testing***

As recommended by the guidelines of CLSI 2019, an antibiotic panel was used for clinical isolates of *P. aeruginosa*. The resistance pattern shown by the different isolates was 45% resistance against amikacin, 60% resistance against ceftazidime, 55% resistance against ciprofloxacin, 58% resistance against cefoperazone/sulbactam, 55% resistance against gentamicin, 51% resistance against meropenem, 53% against imipenem, 40% against piperacillin/tazobactam, 50% against aztreonam, and 45% against cefoxitin. The MAR index for more than 80% of samples was in the range of 0.90–1.00, which was significantly high. 

***Phenotypic detection of MBLs***

Out of a total of 255 isolates of* P. aeruginosa *that were analyzed, 53% were resistant against imipenem. Out of 135 isolates resistant to imipenem, the incidence of MBLs positive was 61.5% (n = 83) as shown by the combination disc test, and 81.5% (n = 110) came out to be producers of MBLs as shown by Modified Hodge Test with imipenem. Modified Hodge test with meropenem reported 75.5% (n = 98) clinical isolates as positive for the production of MBL and 24.5% (n = 32) as negative for MBL production. In total, 65% (n = 87) of clinical isolates were detected as producers of MBL via epsilometer test (E-test). blaIMP-1 was found to exist in 13% (n = 10) of the MBL producers and blaVIM was detected in 15% (n = 12) of MBL producing isolates. 

***Multiplex PCR for blaOXA, blaTEM, blaSHV, blaIMP, and blaVIM***

**Figure 1:**
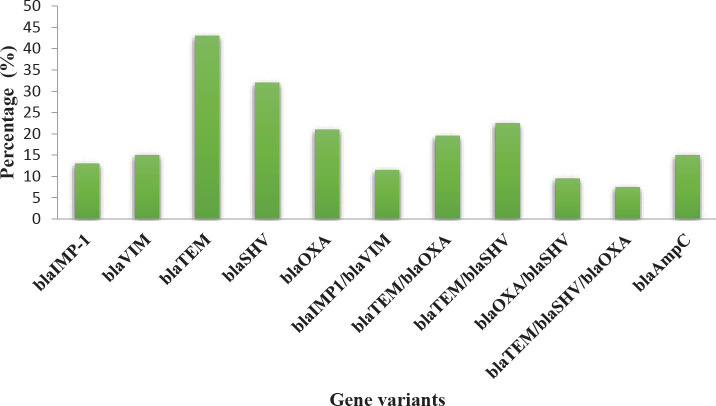
Percentage of gene variants in MBL producing isolates (blaIMP-1, blaVIM), ESBL producing isolates (blaTEM, blaSHV, blaOXA), and blaAmpC

Presence of blaTEM, blaOXA, and blaSHV genes was detected in 52.5% (n = 80) of ESBL-producing strains through multiplex PCR. blaTEM gene was found to exist in 43% of the ESBL producing strains (n = 34), the blaSHV gene was found to exist in 32% (n = 25) and blaOXA gene in 21% (n = 17). blaVIM and blaIMP-1 genes were detected in 11.5% (n = 10) of the MBL-producing strains. Co-existence of these genes was also studied by multiplex PCR. In total, 57.5% (46/80) of the ESBL-positive strains showed co-existence of blaTEM, blaOXA, and blaSHV genes. blaTEM gene co-existed with type blaOXA variants in 19.5% (n = 15) of producers of ESBL, the blaTEM gene co-existed with blaSHV in 22.5% (n = 18) of the strains, blaOXA co-existed with blaSHV in 9.5% (n = 7) and blaSHV, blaTEM and blaOXA co-existed in 7.5% (n = 6) of the ESBL-producing strains. blaAmpC was detected in 15% of the isolates resistant to cefoxitin (n=17) (Figure 1). 

***Statistical analysis***

This was performed by SPSS version 20 for the clinical isolates of *P. aeruginosa *sample-wise, department-wise, and gender-wise by the Chi-square test. *P*-value<0.05 was considered as being statistically significant (Tables 4 and 5). Resistance to imipenem in isolates of *P. aeruginosa* in association with the type of clinical specimen has been tabulated in Table 6.

**Table 1:** Number of patients testing positive in both genders (male, female)

**Total patients**	**Female**	**Male**	**+ve Female**	**+Male**	**Total +ve**	**Total +ve (%)**	**Total ** **-ve**	**Total ** **–ve (%)**
1159	523	636	145	110	255	22%	904	77.9%

**Table 2:** Number of isolates with relation to age groups

**Age groups (years)**	**Total isolates**	**Female**	**Male**
20-29	28	5	23
30-39	57	36	21
40-49	65	55	10
50-59	63	39	24
60-69	42	10	32

**Table 3:** Primers for detection of MBL-type variants (blaIMP-1 & blaVIM), ESBL-type variants (blaSHV, blaTEM & blaOXA) and blaAmpC

**Primer**	**Sequences**	**Annealing temperature (Tm ºC)**	**PCR ** **product**	**Reference**
*bla*_VIM_	ATGGTCGTTATGGCATATC TGGGCCGTGTCAGCCAGAT	57	510	41
*bla*_IMP-1_	AGCGCAGCATATTGATTGC ACAACCAGATGCTGCCTTACC	53.6	587	40
*bla*_SHV_	AGGGCTTGACTGCCATTTTG ATTTGCGTGATTTCATTT	55	400	39
*bla*_TEM_	CCCCGAAGAAGTCCTTTC ATCAGCAATAGTCCCAGC	55	500	39
*bla*_OXA_	ATATCTCGCTTGTTGCATCTCC AAACCCTTCAGCTCATCC	55	600	39
*bla*_AmpC_	CTTCCACACTGCTGTTCGCC -TTGGCCAGGATCACCAGTCC	65	1063	55

MBLs: Metallo-β-lactamases; ESBLs: Extended-spectrum β-lactamase-producing strains

**Table 4:** Isolates of *Pseudomonas aeruginosa* with relation to different wards

**Ward**	**Specimen type**						**Total**
	**Urine**	**Wound**	**Sputum**	**Blood**	**Pus**	**Tissue**	
**Surgery**	30	35	10	9	6	4	94(36.8%)
**Medicine**	26	18	8	7	4	3	66(25.9%)
**Orthopedic**	4	15	7	5	1	2	34(13.3%)
**ICU**	4	15	5	3	1	1	29(11.4%)
**ENT**	3	4	1	2	3	1	14(5.5%)
**Gynecology**	4	2	4	4	3	1	18(7.0%)
**Total**	71	89	35	30	18	12	255(100%)

MBLs: Metallo-β-lactamases; ESBLs: Extended-spectrum β-lactamase-producing strains

**Table 5:** Prevalence of MBL & ESBL producing *Pseudomonas aeruginosa* from clinical samples

**Isolates**	**Urine**	**Wound**	**Sputum**	**Blood**	**Tissue**	**Pus**	***P*** **-value**
blaIMP producing *P. aeruginosa* (n=10)	2	2	1	1	2	2	0.123
blaVIM producing *P. aeruginosa* (n=12)	3	2	1	1	3	2	0.048
blaSHV producing *P. aeruginosa* (n=25)	4	8	9	4	0	0	0.01
blaTEM producing *P. aeruginosa* (n=34)	12	6	10	6	0	0	0.005
blaOXA producing *P. aeruginosa* (n=17)	5	3	2	7	0	0	0.004
blaAmpC producing *P. aeruginosa* (n=17)	6	4	5	2	0	0	0.265
Total	32 (45%)	25 (28%)	28 (80%)	21 (70%)	5 (41%)	4 (22%)	

MBLs: Metallo-β-lactamases; ESBLs: Extended-spectrum β-lactamase-producing strains

**Table 6:** Imipenem resistant isolates in association with the type of clinical specimen

**Isolate**	**Sample**	**(N)**	**Imipenem**		**Chi-square value**	***P*** **-value**
			**S**	**R**		
*Pseudomonas aeruginosa*	Urine	71	17	54	93.14	0.000
	Wound	89	34	55		
	Sputum	35	34	1		
	Blood	30	29	1		
	Tissue	18	2	16		
	Pus	12	4	8		

## Discussion

Empirical drug therapy and over the counter usage of antimicrobial agents is common practice in Pakistan. Unacquainted health care workers, bad sanitation, and unclean conditions in hospital and clinical settings paves way for the pathogens to cause disease. Initially, second-generation antimicrobial drugs were used which have been replaced by the latest drugs and this is becoming a threat to humankind. According to an estimate, the first-line antibiotics have been reported to annually account for approximately 25,692 neonatal deaths in this part of the world (42). Various reports state that resistance against carbapenems has gone up the notch quite significantly especially in Asian countries including India and Pakistan (43, 44). Currently, various reports mention an increasing number of ESBL and MBL producing strains in Pakistan and this heralds an alarming deteriorating situation (45, 46). This study shows the increased frequency of clinical isolates of* P. aeruginosa *being resistant to imipenem plus incidence of MBL producing strains that are associated with resistance to imipenem. A total of 81.5% of the isolates of *P. aeruginosa *that were imipenem resistant were found to be producers of MBL through the Modified Hodge test, showing a lowered incidence in comparison to a study by Shan *et al*. that reported rate of incidence as 87.5% (46). This study shows 53% resistance of *P. aeruginosa *to imipenem which is considerably higher as compared with data reported previously from different Asian countries in the past 10 years (2002–2012) (4). Yet another report stated only 1.9% of resistance against imipenem plus only 2.4% of resistance against meropenem (4). *P. aeruginosa *is the second most prevalent pathogen that has been studied to be in association to resistance against imipenem (46, 47). The frequency of resistance to imipenem as reported from Pakistan was previously 13.42% in 2011 and 28% plus 49.5% in 2015; this demonstrates a significant augmented resistance to imipenem (46, 47, 48). *P. aeruginosa *is amongst the foremost and increasingly prevalent microbes that cause infections in post-burn patients (49, 50). The current study reports blaTEM variants to be majorly in association with resistance against imipenem. All isolates that were found to have blaIMP also had blaTEM. Furthermore, these isolates were studied for any coexistence of MBL and ESBL type variants. 11.5% isolates were detected to co-produce blaIMP-1 and blaVIM genes. One previous report states that there is no significant relationship between MBL and ESBL genes (51). Enhanced production of AmpC-β-lactamases has a negative effect on the outer membrane channels in bacteria that are the route of entry of antimicrobial drugs (52). Antibiotic resistance in *P. aeruginosa *is mainly by virtue of three basic mechanisms that are intrinsic resistant through the presence of AmpC-β-lactamases, MexXY-OprM, and MexAB-OprM efflux pumps, second is acquired resistance through overexpression of AmpC-β-lactamases plus Mex efflux pump system because of mutations occurring in regulatory factors and thirdly the adaptive resistance which is an unstable resistance because of sustained antibiotic pressure (52–54). 15% of the isolates in this study expressed the blaAmpC gene. AmpC-β-lactamases confer high resistance against β-lactam antibiotics (54). Isolates in the current study have shown expression of blaVIM as has been reported in isolates of *P. aeruginosa* in a study conducted in Rawalpindi, Pakistan (55). Coexisting MBL and ESBL genes in the clinical isolates in this study indicate the simultaneous expression of differing variants of MBL/ESBLs in *P. aeruginosa *in the current hospital setting. The current report states that resistance against imipenem in clinical isolates is due to the enzymes of MBL and ESBL types. This report accounts as the first from Pakistan that reports the coexistence of blaIMP, blaVIM, blaTEM, blaSHV, and blaAmpC-type gene variants. No isolate co-exhibited all tested genes (blaIMP-1, blaVIM, blaOXA, blaSHV, blaTEM, and blaAmpC). 

## Conclusion

MBL and ESBL producing variants of *P. aeruginosa *have been found to emerge quite increasingly in this part of the world. A large number of bacterial isolates that were carbapenem-resistant have also demonstrated resistance to the majority of the antibiotics exhibiting augmented pan-resistance to antimicrobial treatment. Determination of the various mechanisms of resistance is very essential. It is imperative to screen isolates for MBLs and ESBLs in the laboratory before commencement of antimicrobial therapy. Further research projects are essentially required for specification of gene variants that show prevalence in clinical isolates in this region of the globe along with the implication of medicine in hospital and healthcare settings. 

## Authors’ Contributions

MM, AR, SSA, and SZHN Study conception and design; MM, SM, and AR Data processing, collection, and performing experiments; AB, SSA, MA, and UF Analysis and interpretation of results; MM, AB, and SZHN Draft manuscript preparation and visualization; MM, SSA, and AB Critical revision and editing of the article; MM and SZHN Final approval of the version to be published; SSA and SZHN Supervision and funding acquisition. 

## Funding

This research did not receive any specific grant from funding agencies in the public, commercial, or not-for-profit sectors. No funding was received for this study. 

## Availability of Data

The data sets analyzed during the current study are available from the corresponding author. 

## Declaration

This study is part of the PhD thesis of Maria Muddassir. 

## Ethics Approval and Consent to Participate

This study was approved by the Ethical Committee of University of Lahore (Ref # IMBB/UOL/20/138). 
